# Assessment and Management of Dermatological Issues in Hospitalized Geriatric Patients: A Retrospective Study in a Tertiary Care Hospital

**DOI:** 10.7759/cureus.75665

**Published:** 2024-12-13

**Authors:** Dilek Menteşoğlu, Selda Pelin Kartal

**Affiliations:** 1 Department of Dermatology and Venereology, Ankara Etlik City Hospital, Ankara, TUR

**Keywords:** aging, consultation, dermatologist, geriatric dermatology, hospitalized patients

## Abstract

Background: Skin problems, typically overlooked in elderly patients hospitalized for systemic diseases, can no longer be ignored.

Objectives: This study aimed to investigate the presence and management of dermatological problems in hospitalized elderly patients.

Materials and methods: This retrospective study involved dermatology consultations for 712 elderly patients (aged ≥ 65 years) hospitalized between October 2022 and October 2023.

Results: The departments that requested the most consultations were physical therapy and rehabilitation (n = 108, 15.2%), internal medicine (n = 64, 9%), and pulmonary disease (n = 52, 7.3%). Consultations were most frequently requested between the seventh and 14th day of hospitalization. The most common reasons for consultation were dermatitis (n = 185; 26%), fungal infections (n = 164; 23%), bacterial infections (n = 54; 7.6%), cutaneous drug reactions (n = 45; 6.3%), and viral infections (n = 44; 6.2%). The most common dermatological diagnoses were xerotic eczema, maculopapular drug eruptions, tinea cruris, tinea ungium, tinea pedis, contact dermatitis, cellulitis, shingles, and xerosis cutis.

Conclusion: These results underscore the importance of convenient access to outpatient services for elderly patients and the need for primary healthcare facilities to have staff trained in geriatric dermatology to ensure optimal care for the aging population.

## Introduction

Over time, individuals age due to internal and external factors, resulting in a decrease in the essential structural elements of the skin, such as lipid content and filaggrin [1]. This decrease affects the protective function of the epidermis against external factors, making it less resistant to infectious agents, leading to issues such as dryness [1]. As the global population ages, doctors encounter more patients aged ≥ 60 years. The effects of new drugs and polypharmacy in the elderly require specific treatment approaches [2]. This has led to the development of a new field known as "geriatric dermatology" to address the skin issues experienced by older patients [3].

Dermatology consultation is requested for geriatric patients receiving inpatient treatment. The purpose of the consultation is to promptly detect and identify skin findings related to dermatological and systemic diseases [4]. Knowing skin diseases in elderly patients and understanding how to manage them can significantly enhance the healthcare services provided to this population, ultimately leading to a positive impact on their lives [5].

Multiple skin problems are identified in elderly patients during outpatient visits. However, arranging transportation for these patients to visit a dermatologist can be challenging because of their advanced age. When an elderly patient is hospitalized for internal or surgical issues, it is crucial not to overlook skin problems. Therefore, our goal was to investigate why various departments request dermatology consultations to improve elderly patient care services and to provide insights into approaches to geriatric dermatology.

## Materials and methods

In this study, we retrospectively analyzed the data of dermatology consultations from 712 elderly patients (aged ≥ 65 years) hospitalized between October 2022 and October 2023. Consultations requested from the emergency departments as well as those with missing data were excluded from the study. Demographic information, medical history, department requesting consultation, time of consultation request (day of hospitalization), reason for consultation, consultation diagnoses, diagnostic methods used during evaluation, and recommended treatments were documented for all patients.

Consultation diagnoses were evaluated with a focus on various categories, including viral infections, bacterial infections, fungal infections, scabies, cutaneous malignancies, seborrheic keratosis, actinic keratosis, dermatitis, xerosis, cutis, pruritus, papulosquamous diseases, immunobullous diseases, adverse drug reactions, urticaria, vasculitis, and others. The study was approved by the Ethics Committee (no. 2024-536), and the requirement for patient consent was waived because this was a retrospective study. This study was conducted in accordance with the ethical principles of the Declaration of Helsinki.

Statistical analysis for this study was conducted using Jamovi (Version 2.3.28, Computer Software, Sydney, Australia). Descriptive statistics were used to summarize the data. Tables displaying the mean ± standard deviation, median, minimum, and maximum values for continuous variables (numeric) are presented. Categorical variables were summarized as numbers and percentages. We conducted Kolmogorov-Smirnov and Shapiro-Wilk tests to assess the normality of the numerical variables.

## Results

Out of the total number of patients, 367 (51.5%) were female and 345 (48.5%) were male. The mean age of the patients was 75.0 ± 7.64 years (Table 1).

**Table 1:** Demographic characteristics of all patients and consultation-related data. ^*^A descriptive statistical test was performed to obtain N and % values. No p-value was used because no comparison was made.

Demographic characteristics and consultation-related data	Mean ± SD, N (%)*
Age	75.0 ± 7.64
Gender	
Female	367 (51.5%)
Male	345 (48.5%)
Age category	
65-69	168 (23.6%)
70-74	176 (24.7%)
75-79	145 (20.4%)
80-84	110 (15.4%)
85-89	67 (9.4%)
90-94	38 (5.3%)
95-100	7 (1%)
>100	1 (0.1%)
Comorbidity	
Hypertension	410 (57.6%)
Type 2 diabetes mellitus	294 (41.3%)
Coronary artery disease	179 (25.1%)
Heart failure	141 (19.2%)
Season in which consultation is requested	
Spring	230 (32.3%)
Summer	235 (33%)
Autumn	42 (5.9%)
Winter	205 (28.8%)
The day of consultation (hospitalization)	
Day 1	57 (8%)
Day 2	122 (17.1%)
Day 3	72 (10.1%)
4-6 days	148 (20.8%)
7-14 days	199 (27.9%)
>2 weeks	114 (16%)

The most common comorbidities among the patients were hypertension (n = 410, 57.6%), type 2 diabetes mellitus (n = 294, 41.3%), coronary artery disease (n = 179, 25.1%), and heart failure (n = 141, 19.8%) (Table 1). Dermatology consultations were mainly requested by the internal medical departments (n = 460, 64.6%). The departments that required the most consultations from dermatology were physical therapy and rehabilitation, internal medicine, and pulmonary diseases (Table 2). The most common diagnoses in consultations in these departments were xerotic eczema, seborrheic dermatitis, tinea cruris, cellulitis, friction dermatitis, oral candidiasis, and xerosis cutis.

**Table 2:** Detailed data on departments requesting consultation. ^*^A descriptive statistical test was performed to obtain N and % values. No p-value was used because no comparison was made.

Departments requesting dermatology consultation	N (%)*
Internal medical departments	460 (64.6%)
Physical therapy and rehabilitation	108 (15.2%)
Internal medicine	64 (9.0%)
Pulmonary diseases	52 (7.3%)
Cardiology	47 (6.6%)
Neurology	34 (4.8%)
Hematology	31 (4.4%)
Nephrology	25 (3.5%)
Infectious diseases	24 (3.4%)
Palliative care	24 (3.4%)
Endocrinology	16 (2.2%)
Oncology	13 (1.8%)
Gastroenterology	13 (1.8%)
Geriatrics	5 (0.7%)
Rheumatology	2 (0.3%)
Psychiatry	2 (0.3%)
Surgical medical departments	141 (19.8%)
Plastic surgery	24 (3.4%)
Orthopedics	23 (3.2%)
Urology	22 (3.1%)
Neurosurgery	19 (2.7%)
General surgery	15 (2.1%)
Cardiovascular surgery	9 (1.3%)
Otorhinolaryngology	7 (1.0%)
Chronic wound care	7 (1.0%)
Obstetrics and gynecology	4 (0.6%)
Surgical oncology	4 (0.6%)
Thoracic surgery	2 (0.3%)
Ophthalmology	2 (0.3%)
Transplantation	2 (0.3%)
Intensive care units	111 (15.6%)
Internal medicine	38 (5.3%)
Surgery	28 (3.9%)
Anesthesia	23 (3.2%)
Neurology	10 (1.4%)
Pulmonary diseases	6 (0.8%)
Coronary	6 (0.8%)

Consultations were primarily requested between the 7th and 14th days of hospitalization. The most common reasons for consultation were dermatitis (n = 185; 26%), fungal infections (n = 164; 23%), xerosis cutis (n = 67; 9.4%), bacterial infections (n = 54; 7.6%), cutaneous drug reactions (n = 45; 6.3%), and viral infections (n = 44; 6.2%) (Table 3).

**Table 3:** Data on consultation diagnoses and diagnostic methods. ^*^A descriptive statistical test was performed to obtain N and % values. No p-value was used because no comparison was made.

Consultation diagnoses and diagnostic methods	N (%)*
Consultation reasons	
Dermatitis	185 (26%)
Fungal infections	164 (23%)
Xerosis cutis	67 (9.4%)
Bacterial infections	54 (7.6%)
Cutaneous drug reactions	45 (6.3%)
Viral infections	44 (6.2%)
Cutaneous malignancies	27 (3.8%)
Generalized pruritus	26 (3.7%)
Urticaria	24 (3.4%)
Scabies	18 (2.5%)
Seborrheic keratosis	16 (2.2%)
Cutaneous vasculitis	8 (1.1%)
Actinic keratosis	7 (1.0%)
Autoimmune bullous diseases	7 (1.0%)
Papulosquamous diseases	6 (0.8%)
Other diseases	111 (15.6%)
Diagnostic methods	
Clinical diagnosis	496 (69.7%)
Clinical diagnosis + Ultrasonography* + Other*	54 (7.6%)
Clinical diagnosis + Other*	45 (6.3%)
Clinical diagnosis + Dermatoscopy	36 (5.1%)
Clinical diagnosis + Punch biopsy	30 (4.2%)
Clinical diagnosis + Ultrasonography*	16 (2.2%)
Clinical diagnosis + Punch biopsy + Dermatoscopy	14 (2.0%)
Clinical diagnosis + Punch biopsy + Direct immunofluorescence	8 (1.1%)
Clinical diagnosis + Punch biopsy + Ultrasonography*+ Other*	5 (0.7%)
Clinical diagnosis + Punch biopsy + Direct immunofluorescence+ Other*	4 (0.6%)
Clinical diagnosis + Punch biopsy + Other*	2 (0.3%)
Clinical diagnosis + Punch biopsy + Ultrasonography*	2 (0.3%)

The most common dermatological diagnoses were xerotic eczema, maculopapular drug eruption, tinea cruris, tinea unguium, tinea pedis, contact dermatitis, cellulitis, shingles, and xerosis cutis (Table 4).

**Table 4:** List of the 50 most common diagnoses during consultations. ^*^A descriptive statistical test was performed to obtain N and % values. No p-value was used because no comparison was made.

Most common diagnoses	N (%)*
Xerotic eczema	59 (8.3%)
Maculopapular drug eruption	40 (5.6%)
Tinea cruris	33 (4.6%)
Tinea ungium + Tinea pedis	32 (4.5%)
Contact dermatitis	31 (4.4%)
Cellulite	29 (4.1%)
Shingles	28 (3.9%)
Xerosis cutis	25 (3.5%)
Stasis dermatitis	24 (3.4%)
Frictional dermatitis	21 (2.9%)
Acute urticaria	20 (2.8%)
Scabies	19 (2.7%)
Xerosis cutis + Pruritus	18 (2.5%)
Herpes infection	16 (2.2%)
Decubitus ulcer	15 (2.1%)
Oral candidiasis	15 (2.1%)
Cutaneous candidiasis	13 (1.8%)
Basal cell carcinoma	13 (1.8%)
Seborrheic keratosis	11 (1.5%)
Seborrheic dermatitis	10 (1.4%)
Tinea ungium	8 (1.1%)
Leukocytoclastic vasculitis	7 (1.0%)
Spontaneous ecchymosis	7 (1.0%)
Tinea pedis+ Tinea ungium+ Cellulite	7 (1.0%)
Nummular dermatitis	6 (0.8%)
Diaper dermatitis	6 (0.8%)
Tinea pedis + Cellulite	6 (0.8%)
Tinea pedis+T inea ungium+ Xerosis cutis	6 (0.8%)
Traumatic bullae	6 (0.8%)
Actinic keratosis	6 (0.8%)
Non-specific dermatitis	6 (0.8%)
Bullous pemphigoid	5 (0.7%)
Tinea pedis + Tinea ungium + Callus	5 (0.7%)
Cutaneous metastasis	5 (0.7%)
Tinea cruris + Diaper dermatitis	4 (0.6%)
Rosacea	4 (0.6%)
Acquired perforating dermatosis	4 (0.6%)
Stasis dermatitis +Xerosis cutis	4 (0.6%)
Generalized pruritus	4 (0.6%)
Drug reaction with eosinophilia and systemic symptoms	4 (0.6%)
Pityriasis versicolor	3 (0.4%)
Prurigo nodularis	3 (0.4%)
Petechiae	3 (0.4%)
Tinea pedis	3 (0.4%)
Psoriasis	3 (0.4%)
Pigmented purpuric dermatosis	3 (0.4%)
Squamous cell carcinoma	3 (0.4%)
Tinea pedis + Tinea ungium + Stasis dermatitis	3 (0.4%)
Venous leg ulcer	3 (0.4%)
Onychogryposis	3 (0.4%)

Approximately 70% of the patients (n = 496) were diagnosed clinically, while the remaining patients underwent additional diagnostic tools such as punch biopsy, dermatoscopy, and ultrasonography (superficial or Doppler) (Table 3). Treatment was recommended for 683 patients (95.9%). The most frequently recommended treatments were topical steroids (n = 269, 37.8%), moisturizers (n = 180, 25.3%), topical antifungals (n = 170, 23.9%), and antihistamines (n = 133, 18.7%) (Table 5).

**Table 5:** Recommended treatments in consultations. ^*^A descriptive statistical test was performed to obtain N and % values. No p-value was used because no comparison was made.

Recommended treatments	N (%)*
Treatment	683 (95.9%)
No treatment	29 (4.1%)
Recommended treatments	
Topical steroids	269 (37.8%)
Topical emollients	180 (25.3%)
Topical antifungals	170 (23.9%)
Antihistamines	133 (18.7%)
Topical antibiotics	85 (11.9%)
Repair barrier creams	58 (8.1%)
Wet dressings	49 (6.9%)
Systemic antibiotics	48 (6.7%)
Systemic antifungals	38 (5.3%)
Systemic antivirals	31 (4.4%)
Systemic steroids	30 (4.2%)
Surgical excision	23 (3.2%)
Topical antiparasitic	22 (3.1%)
Cryotherapy	18 (2.5%)
Topical keratolytic	17 (2.4%)
Systemic antiparasitic	6 (0.8%)
Topical chondroitin polysulfate	6 (0.8%)
Pregabalin	1 (0.1%)
Topical acne medications	1 (0.1%)
Drainage	1 (0.1%)

## Discussion

As the skin is a visible organ, skin problems often prompt individuals of all ages to seek treatment at outpatient dermatology clinics, regardless of their significance [6]. Defining the characteristics of geriatric patients who require dermatological consultations is essential. This helps to identify the unique needs of this patient group, which necessitates specific care and approaches in providing health services [7,8]. 

Although dermatology consultations requested for inpatients are often not prioritized, those requested for hospitalized patients are essential for patient care and can be a significant burden for dermatologists [9]. Cuenca-Barrales et al. showed that approximately 90% of dermatology services provided to hospitalized patients are through consultations requested for inpatients outside the dermatology service [10]. A previous study revealed that most patients in the dermatology department were diagnosed with autoimmune bullous disease and psoriasis. In contrast, most patients seen in the consultations were diagnosed with bacterial infections. The plastic surgery, nephrology, and rheumatology departments requested the most consultations [8]. In our study, internal medical science departments requested approximately 65% of consultations (n = 460). The three departments that requested the most consultations were physical therapy and rehabilitation (n = 108), internal medicine (n = 64), and pulmonary disease (n = 52). We believe consultations are most frequently requested from the physical therapy and rehabilitation department, located in a separate building with a large patient healthcare capacity. Studies have shown that internal medicine is the department that most frequently requests dermatology consultations. This is believed to be because internal medicine serves a larger number of patients for patient care than other departments [11].

In a study on this subject, dermatology consultations of inpatients (n = 429) in a tertiary hospital were analyzed for one year. The average age of the patients was 52 years, and approximately 39% were immunosuppressed. In this study, 80% of consultations were requested from internal medical departments, followed by surgical medical departments (12%), emergency departments (8%), and intensive care units. Within the internal medical departments, the units requesting consultations were internal medicine, hematology, nephrology, and infectious diseases (in decreasing order). Approximately 60% of the consultations were for inflammatory and infectious skin diseases, most dermatitis [12].

A study analyzing referrals to a tertiary hospital revealed that half of the patients were aged > 60 years. The study found that many misdiagnoses were attributed to common skin diseases such as dermatitis, cutaneous infections, and drug eruptions. Additionally, the study found that the dermatitis group accounted for over 50% of referrals. These results emphasize the importance of dermatology departments in general medicine [13]. Similarly, our study's consultations mainly involved dermatitis, fungal infections, xerosis cutis, bacterial infections, and cutaneous drug reactions. The reason for this is that elderly patients are more susceptible to pruritus, dermatitis, and various skin infections because the skin barrier weakens with age, leading to changes in the skin structure [14]. Therefore, regardless of whether the patient is an inpatient or outpatient, most consulted and seen problems are inevitably the same.

A study analyzing dermatology consultations found that two-thirds of the examined patients were clinically diagnosed. The most frequently requested diagnostic tests were skin biopsy, cultures for infectious agents, and the Tzanck smear [15]. Our study found that ultrasonography (superficial or Doppler) was significantly utilized in addition to other diagnostic tests. Ultrasound was requested to rule out deep vein thrombosis and investigate underlying conditions such as venous insufficiency. This approach is essential because edema and venous insufficiency are common in stasis dermatitis, which can sometimes be mistaken for cellulitis [16]. This possibility should not be ignored, especially because elderly hospitalized patients have limited mobility.

Rapid diagnosis and treatment of geriatric patients shorten their hospital stay and contribute to the provision of cost-effective services [17]. In our study, consultations were most frequently requested between the seventh and 14th days (27.9%) and the fourth and sixth days (20.8%) of hospitalization. These periods are considered late because most consultation content was not urgent. This situation implies that patients may have had skin complaints in the initial days of hospitalization, but these concerns may not have been deemed necessary. A study analyzing the content of dermatology consultations revealed that many skin diseases were present before hospitalization and could have been treated through outpatient care [11].

When examining the applications of patients aged > 60 years in the dermatology clinic of a tertiary hospital (n = 457), it was discovered that nearly half of the patients had at least one systemic disease. Hypertension and diabetes are the two most prevalent systemic diseases. Pruritus was most commonly reported by patients with diabetes, whereas eczema was the most prevalent among those with hypertension. Fungal infections were common in both groups [18]. Similarly, the most common comorbidities in the patients in our study were hypertension (57.6%), type 2 diabetes mellitus (41.3%), coronary artery disease (25.1%), and heart failure (19.8%).

Studies have shown that dermatitis, pruritus, and fungal infections are common in geriatric patients, highlighting the need for increased awareness [19]. One key factor is the use of moisturizers for skin care and for adjusting the temperature and humidity of the environment. Foot care is another vital aspect [19]. Enhancing foot care can aid in reducing prevalent fungal infections, such as tinea pedis and tinea unguium, as well as lowering the occurrence of cellulitis in patients [19]. In our study, the most common fungal infections were tinea pedis and tinea unguium, with cellulitis following in terms of frequency. These results demonstrate that neglecting foot care can result in multiple diseases.

Joseph et al. showed that the majority of treatments recommended by dermatologists in consultations (n=676) were topical (n=361). The most common were topical steroids (54%) and moisturizers (42%). Systemic treatments (n=148) included systemic steroids (15%), antihistamines (11%), and antibiotics (5%), which were most frequently recommended [20]. In our study, 95.9% of the patients received treatment. Similarly, topical steroids, moisturizers, and antifungals were the most frequently recommended topical treatments. Differently, the most commonly recommended systemic treatments included antihistamines, antibiotics, and antifungals.

Limitations

Our study has some limitations. First, data from a single-center tertiary care hospital cannot be generalized to the elderly population. Additionally, focusing solely on inpatients may cause us to overlook important conditions referred from the emergency department. However, we aim for the results of our study to raise awareness and demonstrate that inpatients experience similar skin problems as outpatients. Therefore, geriatric dermatology should be considered regardless of whether patients are hospitalized or receiving outpatient care. Another limitation is that the lack of comparisons according to patient comorbidity status may be insufficient to interpret the potential relationship between the most common dermatological problems observed in dermatology consultations and patient characteristics.

## Conclusions

The importance of training healthcare personnel to meet the needs of an aging population in healthcare systems is evident. This training will help reduce the disproportionate burden of skin diseases in elderly patients, which dermatologists cannot keep up with. Our study revealed that the primary reason for dermatology consultations among hospitalized geriatric patients is typical dermatological conditions that can be diagnosed and treated in primary care settings. Providing additional training to physicians, particularly internal medicine or family physicians, on these common dermatological diseases could positively impact resource allocation and time management within healthcare services, ultimately reducing the need for dermatology consultations.

## References

[1] Uprety S, Paudel S, Thapa P (2021). Pattern of skin diseases in geriatric population: our year-long experience from Nepal. Indian Dermatol Online J.

[2] Marinović B (2018). The mature patient: geriatric dermatology. Clin Dermatol.

[3] Wollina U (2011). Geriatric dermatology. Clin Dermatol.

[4] Kroshinsky D, Cotliar J, Hughey LC, Shinkai K, Fox LP (2016). Association of dermatology consultation with accuracy of cutaneous disorder diagnoses in hospitalized patients: a multicenter analysis. JAMA Dermatol.

[5] Jindal R, Jain A, Roy S, Rawat SD, Bhardwaj N (2016). Skin disorders among geriatric population at a tertiary care center in Uttarakhand. J Clin Diagn Res.

[6] Sen A, Chowdhury S, Poddar I, Bandyopadhyay D (2016). Inpatient dermatology: characteristics of patients and admissions in a tertiary level hospital in eastern India. Indian J Dermatol.

[7] Bale J, Chee P (2014). Inpatient dermatology: pattern of admissions and patients' characteristics in an Australian hospital. Australas J Dermatol.

[8] Mthembu L, Masuka JT, Duze K, Mosam A (2023). The characteristics of dermatology inpatients seen at the quaternary Inkosi Albert Luthuli Central Hospital in Durban, South Africa, over a 5-year period - 2015 - 2020. S Afr Med J.

[9] Madigan LM, Fox LP (2019). Where are we now with inpatient consultative dermatology?: assessing the value and evolution of this subspecialty over the past decade. J Am Acad Dermatol.

[10] Cuenca-Barrales C, de Vega-Martínez M, Descalzo-Gallego MÁ, García-Doval I (2021). Inpatient dermatology: where are we headed? A nationwide population-based study of Spain from 2006 to 2016. J Dtsch Dermatol Ges.

[11] Mancusi S, Festa Neto C (2010). Inpatient dermatological consultations in a university hospital. Clinics (Sao Paulo).

[12] Lorente-Lavirgen AI, Bernabeu-Wittel J, Pulpillo-Ruiz Á, de la Torre-García JM, Conejo-Mir J (2013). Inpatient dermatology consultation in a Spanish tertiary care hospital: a prospective cohort study. Actas Dermosifiliogr.

[13] Tay LK, Lee HY, Thirumoorthy T, Pang SM (2011). Dermatology referrals in an East Asian tertiary hospital: a need for inpatient medical dermatology. Clin Exp Dermatol.

[14] Katoh N, Tennstedt D, Abellan van Kan G (2018). Gerontodermatology: the fragility of the epidermis in older adults. J Eur Acad Dermatol Venereol.

[15] Connolly DM, Silverstein DI (2015). Dermatology consultations in a tertiary care hospital: a retrospective study of 243 cases. Dermatol Online J.

[16] Norman RA (2003). Geriatric dermatology. Dermatol Ther.

[17] Alani A, Sadlier M, Uddin A, Hackett C, Ramsay B, Ahmad K (2017). An analysis of inpatient dermatologic consultations at University Hospital Limerick: inadequate infrastructure leads to acute skin failure. Ir J Med Sci.

[18] Nair PA, Vora R (2015). Association of systemic diseases with cutaneous dermatosis in elderly population: preliminary observation at a rural tertiary care centre. J Family Med Prim Care.

[19] Liao YH, Chen KH, Tseng MP, Sun CC (2001). Pattern of skin diseases in a geriatric patient group in Taiwan: a 7-year survey from the outpatient clinic of a university medical center. Dermatology.

[20] Joseph J, Truong K, Smith A, Fernandez-Penas P (2022). Dermatology inpatient consultations in a tertiary hospital - a retrospective analysis. Int J Dermatol.

